# Tissue-Specific Regulation of Na^+^ and K^+^ Transporters Explains Genotypic Differences in Salinity Stress Tolerance in Rice

**DOI:** 10.3389/fpls.2019.01361

**Published:** 2019-11-01

**Authors:** Juan Liu, Sergey Shabala, Lana Shabala, Meixue Zhou, Holger Meinke, Gayatri Venkataraman, Zhonghua Chen, Fanrong Zeng, Quanzhi Zhao

**Affiliations:** ^1^Collaborative Innovation Center of Henan Grain Crops, Henan Key Laboratory of Rice Biology, Henan Agricultural University, Zhengzhou, China; ^2^Tasmanian Institute of Agriculture, University of Tasmania, Hobart, TAS, Australia; ^3^International Research Centre for Environmental Membrane Biology, Foshan University, Foshan, China; ^4^Plant Molecular Biology Laboratory, M.S. Swaminathan Research Foundation, Chennai, India; ^5^School of Science and Health, Western Sydney University, Penrith, NSW, Australia; ^6^Hawkesbury Institute for the Environment, Western Sydney University, Penrith, NSW, Australia; ^7^Department of Agronomy, College of Agriculture and Biotechnology, Zhejiang University, Hangzhou, China

**Keywords:** root, H^+^-ATPase, potassium, sodium, Na^+^/H^+^ exchanger, reactive oxygen species

## Abstract

Rice (*Oryza sativa*) is a staple food that feeds more than half the world population. As rice is highly sensitive to soil salinity, current trends in soil salinization threaten global food security. To better understand the mechanistic basis of salinity tolerance in rice, three contrasting rice cultivars—Reiziq (tolerant), Doongara (moderately tolerant), and Koshihikari (sensitive)—were examined and the differences in operation of key ion transporters mediating ionic homeostasis in these genotypes were evaluated. Tolerant varieties had reduced Na^+^ translocation from roots to shoots. Electrophysiological and quantitative reverse transcription PCR experiments showed that tolerant genotypes possessed 2-fold higher net Na^+^ efflux capacity in the root elongation zone. Interestingly, this efflux was only partially mediated by the plasma membrane Na^+^/H^+^ antiporter (*OsSOS1*), suggesting involvement of some other exclusion mechanisms. No significant difference in Na^+^ exclusion from the mature root zones was found between cultivars, and the transcriptional changes in the salt overly sensitive signaling pathway genes in the elongation zone were not correlated with the genetic variability in salinity tolerance amongst genotypes. The most important hallmark of differential salinity tolerance was in the ability of the plant to retain K^+^ in both root zones. This trait was conferred by at least three complementary mechanisms: (1) its superior ability to activate H^+^-ATPase pump operation, both at transcriptional and functional levels; (2) reduced sensitivity of K^+^ efflux channels to reactive oxygen species; and (3) smaller upregulation in *OsGORK* and higher upregulation of *OsAKT1* in tolerant cultivars in response to salt stress. These traits should be targeted in breeding programs aimed to improve salinity tolerance in commercial rice cultivars.

## Introduction

Soil salinization is a major abiotic constraint for crop productivity worldwide (Munns and Gilliham 2015; Yang and Guo 2018). About 50% of cultivated lands are estimated to be affected by salinity; this proportion is expected to increase, due to global climatic changes and increased use of low-quality irrigation water for crop production (Pitman and Läuchli, 2004; Shabala, 2013; Suzuki et al., 2016).

Rice (*Oryza sativa* L.) is one of the world’s most important cereal crops accounting for over 20% of all calories in the human diet (Schatz et al., 2014). Rice plants are also highly sensitive to salinity stress, and the progress in breeding rice for salinity tolerance has been rather modest, due to the complex physiological, biochemical, and molecular mechanisms conferring this trait (Ismail and Horie, 2017; Li et al., 2017; Formentin et al., 2018). Salt simultaneously causes osmotic and ion toxicity stress, as well as leads to various plant nutritional disorders and oxidative stress on plants. As a result, depending on their habitat and severity of stress, plants have evolved various strategies to minimize the damage associated with salt. These mechanisms include osmotic adjustment, Na^+^ exclusion and sequestration, and K^+^ retention in the cytosol, control of xylem ions loading, and oxidative stress tolerance (Ashraf et al., 2008; Adem et al., 2014; Bose et al., 2014a; Chakraborty et al., 2016). All of these mechanisms should be considered in the genetic design of a salt-tolerant rice plant.

Na^+^ is a major toxic element in saline-affected soil. Several studies have revealed that salt-tolerant rice cultivars accumulate less Na^+^ in leaves and shoots than that in salt-sensitive rice cultivars (Golldack et al., 2003; Lee et al., 2003; Lin et al., 2004; Ren et al., 2005; Cotsaftis et al., 2011). Thus, it is not surprising that the main thrust of breeders has been directed towards the traits enabling Na^+^ exclusion from the shoot (Fukuda et al., 2004; Anil et al., 2007; Kavitha et al., 2012). At the cellular level, Na^+^ exclusion from the root uptake is mediated by the gene salt overly sensitive 1 (SOS1) that encodes plasma membrane (PM)-localized Na^+^/H^+^ exchanger (Horie et al., 2009; Henderson et al., 2015) in root epidermis. The operation of SOS1 Na^+^/H^+^ exchanger is driven by a proton gradient generated by PM H^+^-ATPase (Blumwald et al., 2000). SOS1 gene is also found to be expressed in the root stele (Shi et al., 2002), where it may mediate xylem Na^+^ loading (Zhu et al., 2016; Zhu et al., 2017). El Mahi et al. (2019) reported that OsSOS1 regulates both net root Na^+^ uptake and long-distance Na^+^ transport to shoots in rice. This causes a potential dilemma, as any attempt to enhance Na^+^ extrusion to the rhizosphere by increasing SOS1 transcript level may come with a caveat of increasing xylem Na^+^ loading (Ishikawa and Shabala, 2018). An important research question is therefore: how do *O. sativa* handle this dilemma? This is further complicated by the distinct anatomy of the rice root as well as the occurrence of apoplastic bypass flow that plays an important role in mediating Na^+^ movement in rice (Yeo et al., 1987; Ochiai and Matoh, 2002; Krishnamurthy et al., 2011; Faiyue et al., 2012; Flam-Shepherd et al., 2018). Tu et al. (2014) found that salt-tolerant tetraploid rice plants formed less aerenchyma and possessed a thicker barrier in the endodermis, thus blocking deleterious ion transport to pericycle cells, as compared to salt-sensitive diploid rice. In this context, does SOS1-mediated Na^+^ exclusion from cytosol play a major role in rice salinity tolerance?

Superior K^+^ retention in salt-stressed roots is positively correlated with salt tolerance in a larger number of plant species (reviewed in Wu et al., 2018a). Salt stress causes significant membrane depolarization in root cells (by 50–80 mV, depending on stress severity; Shabala et al., 2016 and references therein). This depolarization activates outward-rectifying K^+^-selective (GORK) channels and explains the massive K^+^ loss reported upon acute salinity treatment from roots of several plant species, including *Arabidopsis* (Shabala et al., 2005; Shabala et al., 2006), barley (Chen et al., 2007a; Shabala et al., 2016), *Populus euphratica* (Sun et al., 2009; Chen and Polle, 2010; Zhao et al., 2016), and mangrove species (Lu et al., 2013; Lang et al., 2014). At the same time, Coskun et al. (2013) has concluded that K^+^ efflux is a poor predictor of salt tolerance in rice. However, this conclusion is derived from the use of an indirect method to estimate K^+^ efflux involving radio tracer ^42^K flux analysis that relies on several assumptions related to compartmentation analysis. To the best of our knowledge, no direct evaluation of the role of root K^+^ retention as a component of salinity tissue tolerance mechanism in rice has been conducted.

Salt stress also induces overaccumulation of reactive oxygen species, which causes oxidative stress (Miller et al., 2010). Plant roots harbor a large array of reactive oxygen species (ROS)-activated nonselective cation channels (Demidchik, 2018). These channels are permeable to K^+^ and may represent another major pathway mediating K^+^ efflux under salt stress (Demidchik, 2014, Shabala and Pottosin, 2014). What is the role of ROS-activated K^+^ channels in relation to salinity tolerance in rice?

By depolarizing the plasma membrane, salt stress also reduces, or makes it thermodynamically impossible for K^+^ uptake to occur through inward-rectifying K^+^ channels (Shabala and Cuin, 2008), increasing the role of high affinity K^+^ uptake systems in K^+^ acquisition. In rice, the K^+^ transporter genes *OsHAK1* and *OsHAK5* are induced by salt stress, mediating K^+^ uptake and transport to maintain a high K^+^/Na^+^ ratio under salt stress (Yang et al., 2014; Chen et al., 2015); however, this process is not observed in barley (Shabala and Cuin, 2008; Boscari et al., 2009). These results suggest that rice might possess distinctive pathways for K^+^ homeostatic modulation. What are these pathways, and what role does K^+^ retention play in salinity tolerance in rice?

This study aimed to provide the answers to some of these questions and investigated the relative contribution of each of these described mechanisms to salt tolerance in three contrasting rice cultivars. By combining electrophysiological [a noninvasive microelectrode ion flux estimation (MIFE) technique] and molecular [quantitative reverse transcription PCR (qRT-PCR)] approach, we have explored the cell-type-specific mechanisms underlying differences in responses to salinity stress in these cultivars. Our data suggests that K^+^ retention plays a prominent role in conferring genetic variability in salinity tolerance trait in rice, and is conferred by orchestrated regulation of several mechanisms, both at transcriptional and functional levels.

## Materials and Methods

### Plant Materials, Growth Conditions, and Stress Treatments

Three different rice (*O. sativa*) cultivars, Koshihikari, Doongara, and Reiziq, were used in this study. Koshihikari is a *japonica* genotype, whereas Reiziq and Doongara are Australian commercial *indica* varieties. Seeds were obtained from the Western Sydney University and multiplied in a glasshouse at Tasmanian Institute of Agriculture facilities in Hobart. Seeds were germinated in deionized water in a growth chamber at 28°C in the dark for 2 days. Uniformly germinated seeds were then transferred to the hydroponics system and grown in International Rice Research Institute nutrient solution (Yoshida, 1976) at 28°C/25°C (day/night) under a 14 h/10 h photoperiod for 3 weeks. Seedlings were then exposed to salt stress by adding 0, 50, and 100 mM NaCl to the hydroponic solution. The solution was renewed every 3 days. After 15 days of salinity exposure, plants agronomical characteristics were measured, and plants were sampled for analysis of ion content in the roots and shoots.

### Measurement of Root Length, Plant Height, and Plant Biomass

Root length and plant height were measured using a ruler. Fresh weight (FW) was determined using an electronic balance immediately after cutting. Fresh samples were then washed with deionized water and dried at 70°C to a constant weight (DW). Water content (WC) of each plant was determined as a percentage as WC = (FW − DW)/(FW) × 100.

### Chlorophyll Content and Fluorescence Measurement

Leaf chlorophyll content was quantified using a SPAD-502 chlorophyll meter (Konica Minolta, Osaka, Japan). Measurements were taken from the middle part of the first fully developed leaf. Chlorophyll fluorescence measurements were conducted on the same leaves as Soil–Plant Analyses Development (SPAD), using the OS-30p portable chlorophyll fluorometer (Opti-Science Inc., Tyngsboro, MA, USA). Leaves were dark-adapted for 20 min before the measurements. Initial (F*o*), variable (F*v*), and maximum (F*m*) chlorophyll fluorescence characteristics were recorded. The photochemical efficiency of PSII was then calculated as the ratio of F*v*/F*m*.

### Tissue Na^+^ and K^+^ Content

Dry rice root and shoot samples were ground into powder and digested with 98% H_2_SO_4_–30% H_2_O_2_. The digested samples were diluted with distilled water, and Na^+^ and K^+^ content were determined using a flame photometer (PFP7, Jenway; Bibby Scientific Ltd., Stone, UK). The Na^+^ translocation coefficient from roots to shoots was calculated as Na^+^ content in the root divided by Na^+^ content in the shoot.

### Viability Staining and Anatomical Analysis of Roots

Five-day old rice seedlings were treated with 100 mM NaCl for 48 h, and then the primary roots were sampled and used for viability staining and anatomical analysis. The viability of root cells was determined by using the fluorescein diacetate (FDA)–propidium iodide (PI) double staining method as described by Chakraborty et al. (2016). Root cells viability is represented by the relative intensity of the green fluorescence signal and calculated as: CTCF = integrated density − (selected area × background mean intensity). Root cross-sections were excised at 1.5 mm from the root apex. Roots were sectioned by using a paraffin embedding method and anatomical structure observed by an optical microscope (BX51; Olympus, Tokyo, Japan).

### MIFE Noninvasive Ion Flux Measurements

Net fluxes of H^+^, K^+^, and Na^+^ were measured using the noninvasive microelectrode MIFE technique (University of Tasmania, Hobart, Australia; Shabala et al., 1997). The theory of MIFE measurement and ion-selective microelectrode fabrication and calibration processes have been described previously (Shabala and Shabala, 2002; Shabala et al., 2006). Rice seeds were germinated in Petri dishes with deionized water in a growth chamber at 30°C in the dark. After 2 days, germinated seeds were grown in filter paper rolls set inside containers with basic solution media (BSM: 0.5 mM KCl, 0.1 mM CaCl_2_, 0.2 mM NaCl) for 3 days. Plants with roots length 40–60 mm were used for ion flux measurements. For each treatment, net ion fluxes were measured from the elongation (1200–1500 µm from the root apex; Takehisa et al., 2012) and mature (1.2–1.5 cm from the root apex) zones of at least six individual roots.

### Transient Ion Flux Kinetics

The rice root was immobilized horizontally in a measuring chamber containing 30 ml BSM solution for 30 min prior to measurements, with the shoot in the air and the root–shoot junction below the solution level. Measurements were conducted at room temperature (23 ± 1°C). Net ion flux was first measured for 5 min in BSM solution, to record steady state initial flux values. The appropriate test solution (either 100 mM NaCl or 10 mM H_2_O_2_) was then administered, and transient ion flux responses were measured for another 30 min. H_2_O_2_ (10 mM) was selected as a physiologically relevant treatment (e.g. a concentration typically found in salinized root tissues; Huang et al., 2019) including rice (Feng et al., 2013). A delay of 2 min is caused by the act of replacing the BSM solution in the measuring chamber with the appropriate treatment solution to achieve the non-stirred layer required in MIFE measurements and thus appears as a gap in the graphs. Net fluxes of ions were calculated using MIFEFLUX software from recorded voltage outputs, assuming cylindrical root geometry, as described elsewhere (Shabala et al., 1997).

### Quantification of Na^+^/H^+^ Exchanger Activity in Rice Roots

A so-called “recovery protocol” (Cuin et al., 2011) was used to quantify the operation of PM-based Na^+^/H^+^ exchangers in the root elongation and mature zones. Five-day-old rice seedlings were treated with 100 mM NaCl for 24 h in darkness, to accumulate salt and activate Na^+^ efflux systems. Prior to measurement, the root was quickly and thoroughly rinsed with 10 mM CaCl_2_ solution for 1 min to remove apoplastic NaCl. The root was then transferred into a clean chamber containing Na-free BSM solution and kept for 30 min. Net steady Na^+^ fluxes were then measured in each zone for 3–5 min. As shown previously in pharmacological (Wu et al., 2019) and genetic studies (Cuin et al., 2011), Na^+^ fluxes measured by employing this protocol reflect the functional activity of SOS1-like Na^+^/H^+^ exchangers in the plasma membrane of epidermal root cells.

### Pharmacological Measurements

About 30–60 min prior to 100 mM NaCl or 10 mM H_2_O_2_ treatment, rice roots were pretreated for 1 h with one of the following blockers: 1 mM sodium orthovanadate (vanadate), a known blocker of PM H^+^-ATPase; 1 mM amiloride, an inhibitor of the PM Na^+^/H^+^ exchanger activity; 20 mM TEA, a known blocker of K^+^-selective PM channels. All inhibitors were prepared in background BSM solution.

### Gene Expression Analysis

To determine relative gene expression levels, 5-day-old rice seedlings were exposed to 100 mM NaCl for 0, 1, and 48 h. The root tip (0–2 mm from the root apex), encompassing the root cap, meristem, and elongation zone was excised with a scalpel, and the remaining root system beyond 0–10 mm from the root apex was considered the mature zone (**Figure S1**). Three independent technical replicates, each containing a pool of 10 plants, were sampled for qRT-PCR validation. Total RNA was extracted from roots using TRIzol reagent (Invitrogen, Carlsbad, CA) and reverse-transcribed using MMLV Reverse Transcriptase (Promega, Madison, WI, USA) according to the manufacturer’s instructions. qRT-PCR was performed using GoTaq qPCR Master Mix (Promega, Madison, WI, USA) on a CFX 96 Real Time System RT-PCR system (Bio-Rad, Hercules, CA, USA). The 2^−ΔΔCT^ method was used to determine the relative expression levels of the studied genes, related to SOS pathway, K^+^ channels and transporters, and PM H^+^-ATPase (Livak and Schmittgen, 2001). Three independent biological replicates (each made of 10 individual plants) were used in this experiment. The primers used for qRT-PCR analysis are listed in **Table S1**.

### Statistical Analyses

The IBM SPSS Statistics software was used to perform all statistical analysis. Significant differences between treatments were assessed using Duncan’s multiple range test at a significance level of 0.05 or 0.01. All data in tables and figures are means ± standard error (SE).

## Results

### Morphological Responses of the Three Rice Cultivars to Salt Stress

**Figure 1:**
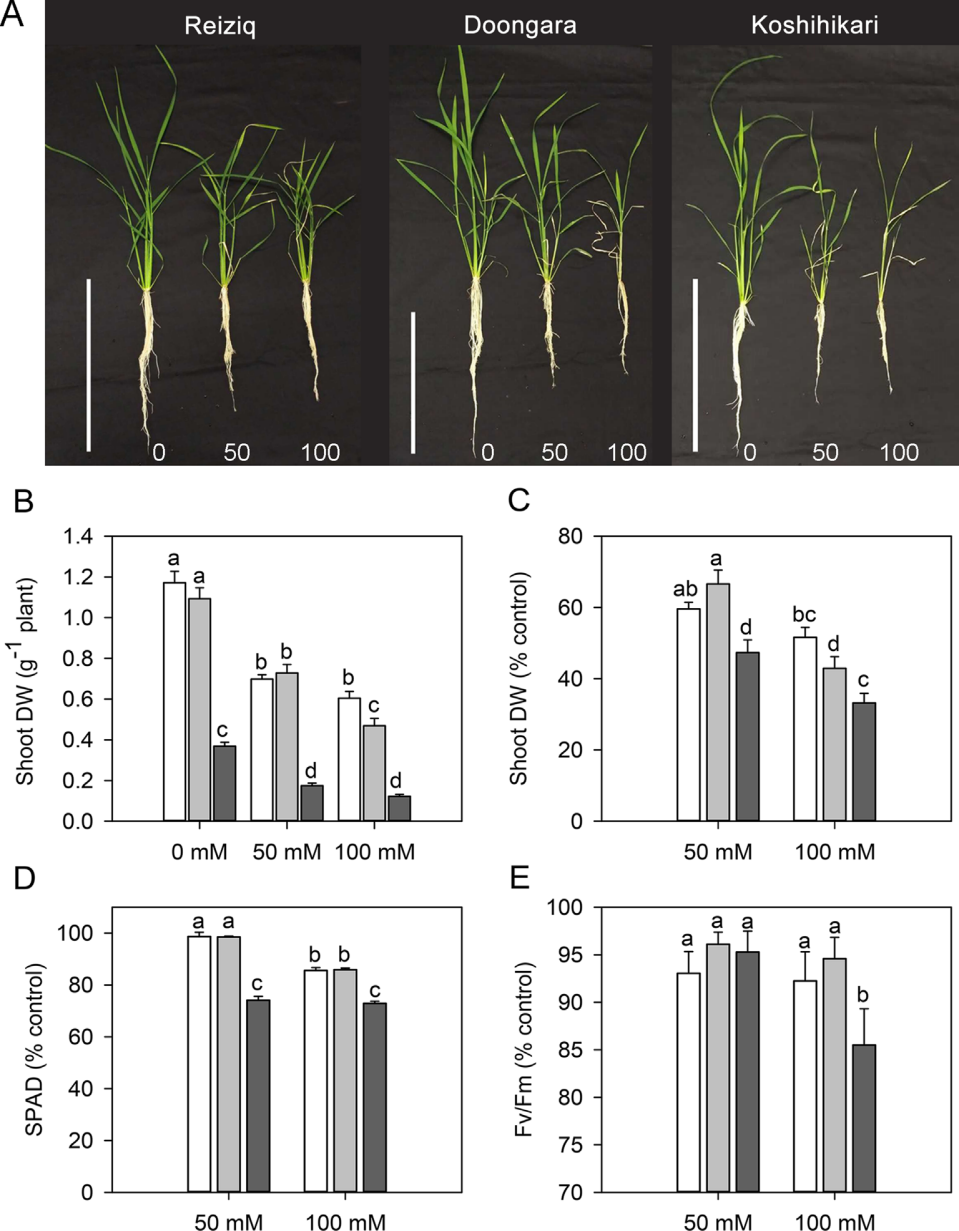
**(A)** Morphological growth differences of three rice cultivars (Reiziq, Doongara, Koshihikari) grown at three salinity levels (0, 50, and 100 mM NaCl) for 15 days, bars = 30 cm. Dry weight of shoot **(B**, **C)**, leaf chlorophyll content [Soil–Plant Analyses Development (SPAD) value; **D**], and chlorophyll fluorescence (F*v*/F*m*; **E**) of three rice cultivars grown at three salinity levels for 15 days. Mean ± SE (*n* = 8). Data labeled by different letters are significantly different at *P* < 0.05.

Salinity stress resulted in a significant reduction in plant height, root length, tiller number, FW and DW of shoot and root, and WC in all cultivars (**Table 1**, **Figure 1**). Cultivar Koshihikari was the most sensitive of all the three, showing 42% and 57% reduction in plant height and tiller number, respectively, when grown in the presence of 100 mM NaCl (**Table 1**). Compared to Koshihikari, Reiziq and Doongara showed lower reduction in shoot DW under salt stress and performed similarly at a moderate concentration of salinity (50 mM); under the more severe treatment (100 mM NaCl), variety Reiziq’s performance was the best (**Figures 1B, C**). Consistent with these findings were measurements of chlorophyll content and efficiency of operation of PSII in salt-grown plants, with SPAD and Fv/Fm parameters being more reduced in Koshihikari compared with other cultivars (**Figures 1D, E**). Taken together, our results suggest that salinity stress tolerance in these cultivars occurs in the order: Reiziq > Doongara > Koshihikari.

**Table 1:** Morphological growth change of three rice cultivars grown at three salinity levels for 15 days.

Plant material	Treatment level	Plant height (cm)	Root length (cm)	Tiller number	Fresh weight of shoot (g plant^−1^)	Fresh weight of root (g plant^−1^)	Dry weight of root (g plant^−1^)	Water content (% w/w)
	0 mM	55.6 ± 1.3^b^	28.2 ± 0.6^b^	4.4 ± 0.2^a^	6.0 ± 0.3^a^	2.9 ± 0.1^a^	0.28 ± 0.02^a^	83.6 ± 0.2^a^
Reiziq	50 mM	35.9 ± 0.3^e^	20.0 ± 0.3^e^	2.9 ± 0.2^bc^	3.1 ± 0.1^b^	1.3 ± 0.04^c^	0.14 ± 0^b^	81.2 ± 0.1^b^
	100 mM	35.8 ± 0.8^e^	20.0 ± 0.4^e^	2.8 ± 0.1^c^	2.3 ± 0.1^c^	1.4 ± 0.1^c^	0.15 ± 0.01^b^	79.2 ± 0.2^d^
	0 mM	59.1 ± 0.6^a^	39.6 ± 0.9^a^	3.2 ± 0.1^b^	5.5 ± 0.3^a^	2.4 ± 0.1^b^	0.26 ± 0.01^a^	82.9 ± 0.1^a^
Doongara	50 mM	48.6 ± 1.8^c^	22.5 ± 0.7^d^	2.8 ± 0.1^bc^	3.5 ± 0.2^b^	1.2 ± 0.1^c^	0.14 ± 0.01^b^	81.5 ± 0.2^b^
	100 mM	43.3 ± 0.3^d^	20.7 ± 0.6^e^	2.5 ± 0.2^c^	1.8 ± 0.2^c^	0.8 ± 0.1^d^	0.09 ± 0.01^c^	78.9 ± 0.2^d^
	0 mM	55.4 ± 1.1^b^	25.7 ± 0.6c	2.8 ± 0.13^bc^	1.9 ± 0.1^c^	0.8 ± 0.04^d^	0.09 ± 0.01^c^	83 ± 0.2^a^
Koshihikari	50 mM	35.6 ± 1.4^e^	16.8 ± 0.5^f^	1.8 ± 0.1^d^	0.8 ± 0.1^d^	0.3 ± 0.02^e^	0.04 ± 0^d^	80.5 ± 0.5^c^
	100 mM	32.3 ± 1.2^f^	15.1 ± 0.6^g^	1.2 ± 0.1^e^	0.5 ± 0.1^d^	0.3 ± 0.03^e^	0.02 ± 0^d^	78.2 ± 0.5^e^

Data are presented as the means ± SE of ten replicates, and significant differences (P < 0.05) between different cultivars and treatments are indicated by different letters.

### Effect of Salt Stress on Na^+^ and K^+^ Content in Roots and Shoots

**Figure 2:**
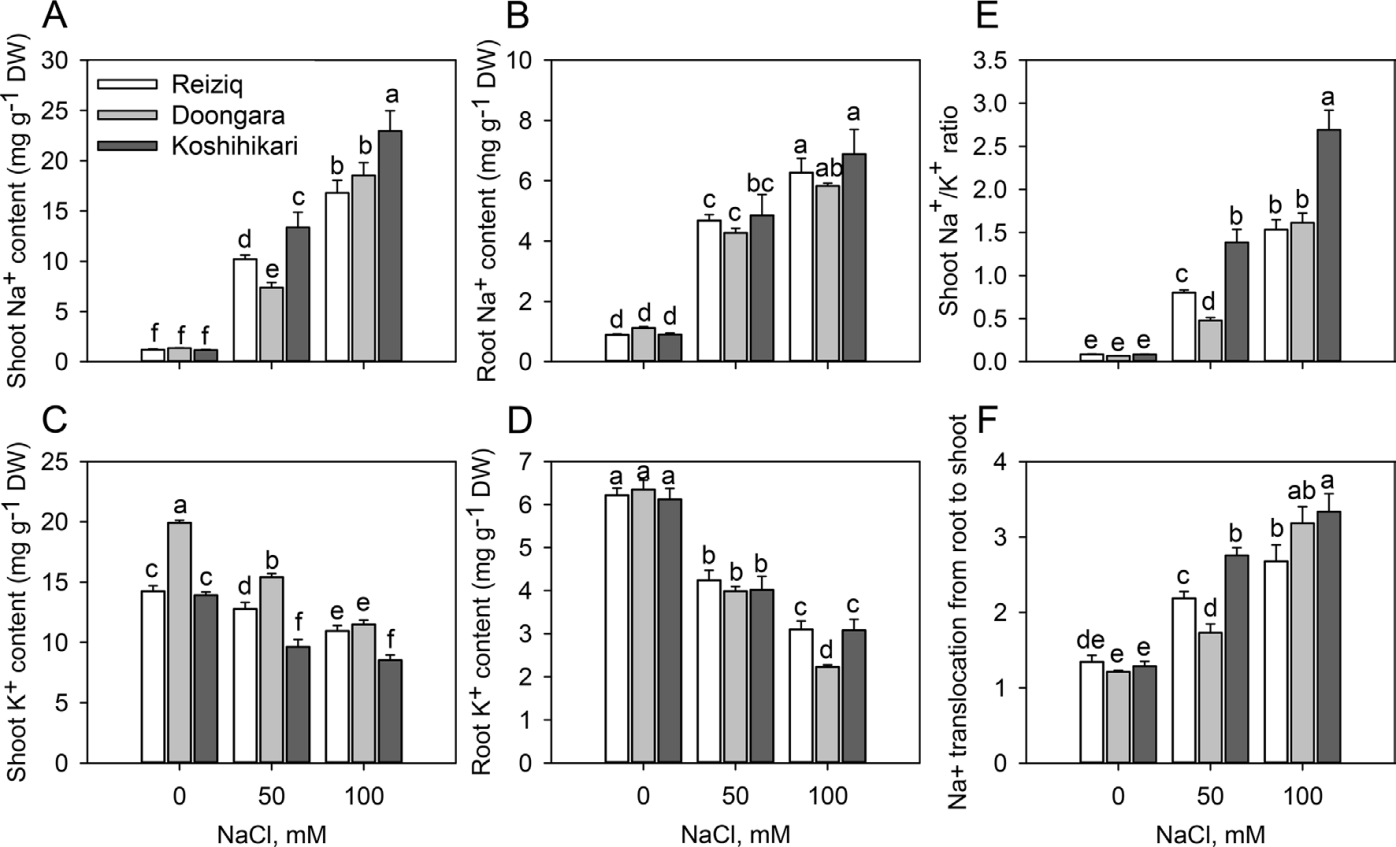
Na^+^ and K^+^ contents in shoots **(A**, **C)** and roots **(B**, **D)**, Na^+^/K^+^ ratio in soots **(E)**, and Na^+^ translocation from roots to shoots **(F)** in the three rice cultivars grown at the three salinity levels for 15 days. Mean ± SE (*n* = 6). Data labeled by different letters are significantly different at *P* < 0.05.

Na^+^ and K^+^ contents were determined in shoots and roots after 15 days of treatment with 50 and 100 mM NaCl (**Figure 2**). In the absence of NaCl, the three rice cultivars showed no differences in Na^+^ or K^+^ contents in roots (**Figures 2A, B, D**); shoot K^+^ content was slightly higher in Doongara (**Figure 2C**). Salt stress significantly increased Na^+^ content, but decreased K^+^ content, in shoots and roots of all the three rice cultivars compared to the nonsalt control (**Figures 2A–D**). Genotypic differences in Na^+^ and K^+^ contents under salt stress were more obvious in shoots than in roots, indicating a significant difference in shoot Na^+^ translocation among these cultivars. After exposure to 50 or 100 mM NaCl, tolerant varieties Reiziq and Doongara accumulated less Na^+^ and maintained higher K^+^ levels in shoots, compared to Koshihikari (**Figures 2A, C**). They also had significantly lower Na^+^/K^+^ ratios in shoots (**Figure 2E**) and lower Na^+^ translocation from roots to shoots, except the difference in Na^+^ translocation from roots to shoots between Doongara and Koshihikari exposed to 100 mM NaCl (**Figure 2F**).

### Effects of Salt Stress on Root Cell Viability and Anatomical Structure in Root Elongation and Mature Zones

**Figure 3:**
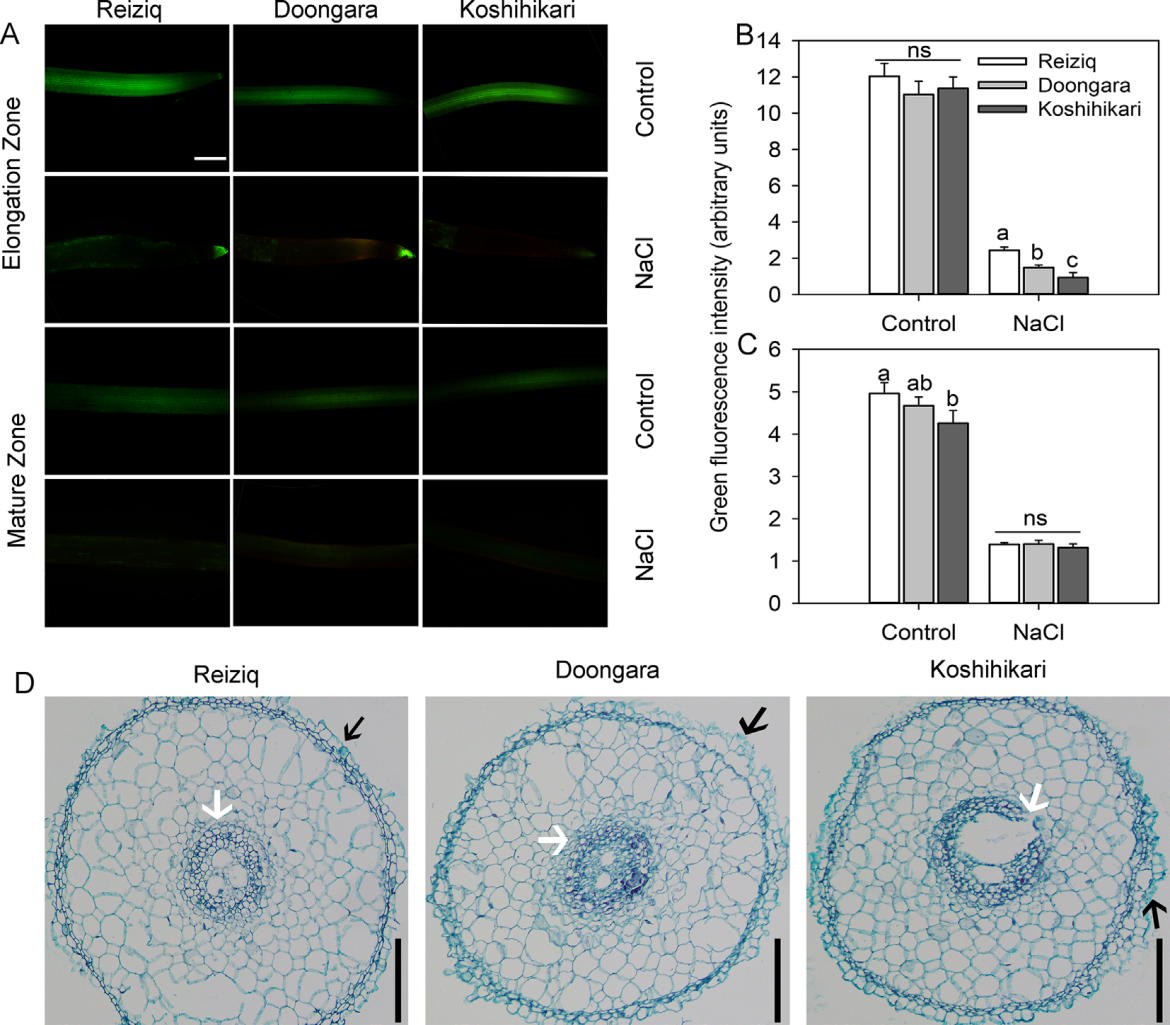
Viability staining of the elongation and mature root zones of the three rice cultivars exposed to 100 mM NaCl for 48 h. **(A)** One typical image is shown for each treatment/cultivar, bars = 100 μm. The intensity of the green fluorescent signal represents the root cell viability in the elongation **(B)** and mature **(C)** zones. Values are the mean ± SE (*n* = 15–20). **(D)** Anatomical structure in the rice root elongation zone (1.5 mm from the root apex) after the treatment of 100 mM NaCl for 48 h, bars = 50 μm. The black arrow indicates exfoliated root epidermis cells. The white arrow indicates a protective gap formed between the cortex cells and pericycle cells. Data labeled by different letters are significantly different at *P* < 0.05; ns, nonsignificant.

To investigate the effects of salt stress on rice root cell characteristics, we determined root viability by FDA/PI double staining method (**Figure 3A**). After 48 h of 100 mM NaCl treatment, there was a strong reduction in the root viability in both mature and elongation root zones. Cells in the elongation zone were more sensitive to salinity, and the extent of damage (as estimated by viability staining) inversely correlated with plant salinity tolerance (e.g. damage in Koshihikari > Doongara > Reziq) (**Figure 3B**). No significant (*P* < 0.05) genotypic differences in cell viability were detected in the mature root zone (**Figure 3C**). Exposure to salinity resulted in shrinkage of the pericycle and exfoliation of the root epidermal cells, and this effect was inversely proportional to salinity stress tolerance (**Figure 3D**).

### Differential Na^+^ Exclusion Capacity in Root Elongation and Mature Zones Under Salt Stress

**Figure 4:**
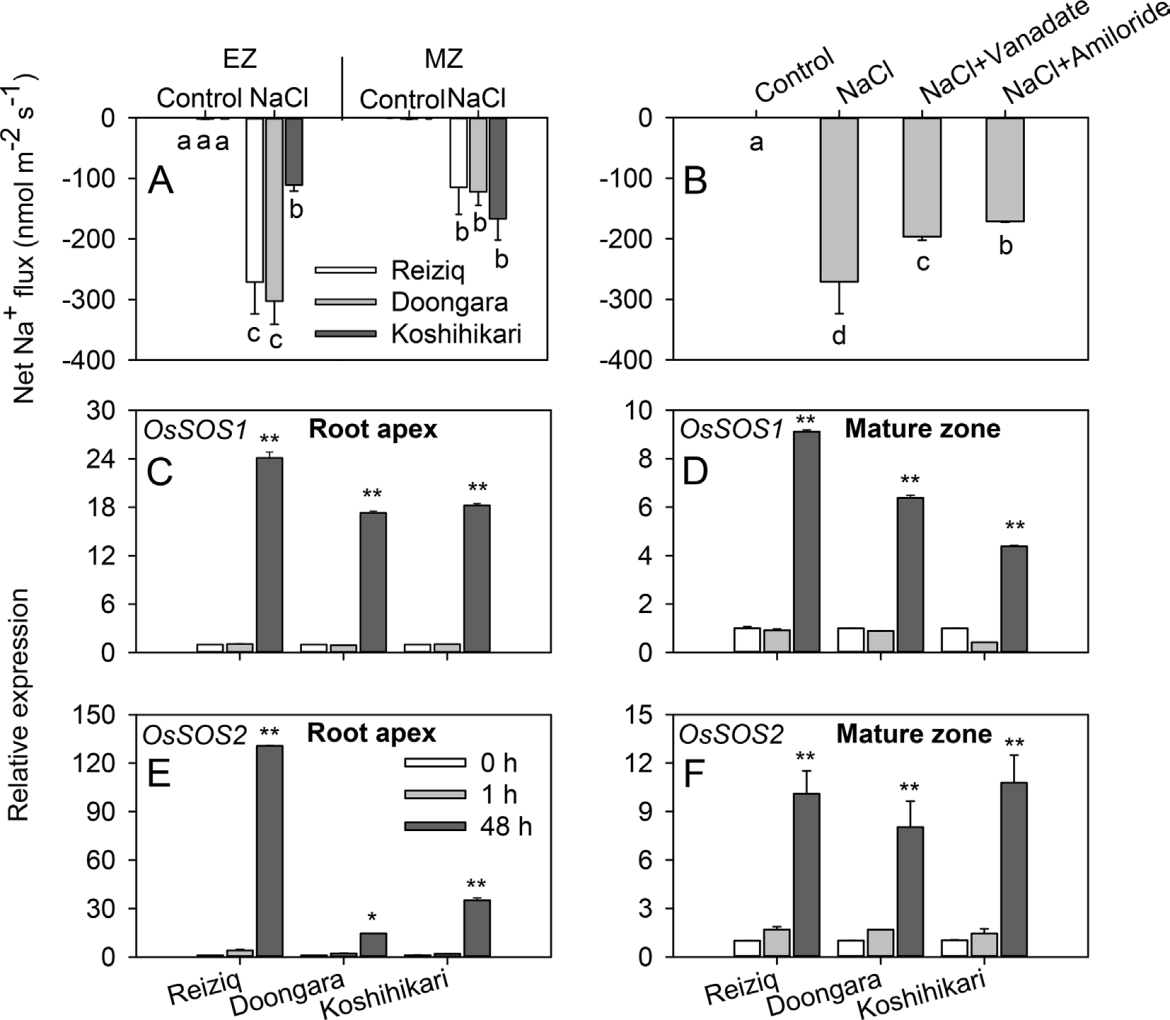
Net Na^+^ effluxes from root elongation and mature zones of the three rice cultivars. **(A)** Roots were exposed to 0 (control) or 100 mM NaCl (NaCl) for 24 h and then transferred to basic salt media (BSM) solution without 100 mM NaCl for 30 min. Net steady-state fluxes were measured for 3–5 min. **(B)** Net Na^+^ effluxes were measured from elongation root zone of cultivar Reiziq without (control) or with inhibitors: 1 mM sodium orthovanadate (vanadate) (a known blocker of the plasma membrane H^+^-ATPase), 1 mM amiloride (an inhibitor of the plasma membrane Na^+^/H^+^ exchanger activity). Rice roots were pretreated with the inhibitors for 1 h. Quantitative reverse transcription PCR (qRT-PCR) analysis of the time-dependent expression pattern of *OsSOS1* **(C**, **D)**, *OsSOS2* **(E**, **F)** in the root tips **(C**, **E)**, and mature zones **(D**, **F)** of the three cultivars. Asterisk * or ** indicate a significant difference between tissues at *P* < 0.05 or *P* < 0.01, respectively.

Salinity stress tolerance is strongly associated with the ability of the plant to exclude Na^+^ from the cytosol (Munns and Tester, 2008). Accordingly, we have compared patterns of net Na^+^ fluxes from the elongation and mature zones of roots of three contrasting cultivars. A significant Na^+^ efflux was measured from epidermal cells in both root zones. In the elongation zone, net Na^+^ efflux was significantly higher in tolerant Reiziq and Doongara compared with salt-sensitive Koshihikari (**Figure 4A**). No significant (at *P* < 0.05) difference in net Na^+^ efflux was found between contrasting cultivars in mature root zone. Pharmacological experiments revealed that Na^+^ efflux in the elongation zone was significantly suppressed by amiloride, an inhibitor of PM Na^+^/H^+^ exchanger activity, and by sodium orthovanadate, a known blocker of PM H^+^-ATPase (**Figure 4B**), although in both cases the block was only partial.

To evaluate the role of SOS pathway in root Na^+^ exclusion, we quantified changes in the expression levels of *OsSOS1* and *OsSOS2* transcripts in the root apex and mature zones. Short-term salt stress did not induce any change in the expression of *OsSOS1* that encodes for PM-localized Na^+^/H^+^ exchanger, whereas long-term (48 h) salinity exposure significantly upregulated *OsSOS1* expression, with *OsSOS1* expression being several folds higher in root tips than in the mature zone (∼20-fold and 6-fold in elongation and mature zones, respectively; **Figures 4C, D**). In both zones, this upregulation was the strongest in the most tolerant cultivar Reiziq (**Figures 4C, D**). *OsSOS2* transcripts were also significantly induced in both the root zones after 48 h of salt treatment (**Figures 4E, F**). Reiziq showed the highest induction (ca. 130-fold) in root tips. In mature zone, salinity-induced increase in *OsSOS2* transcript levels was not significantly different between the cultivars.

### K^+^ Transport in Root Epidermis in Response to NaCl and H_2_O_2_ Treatment

**Figure 5:**
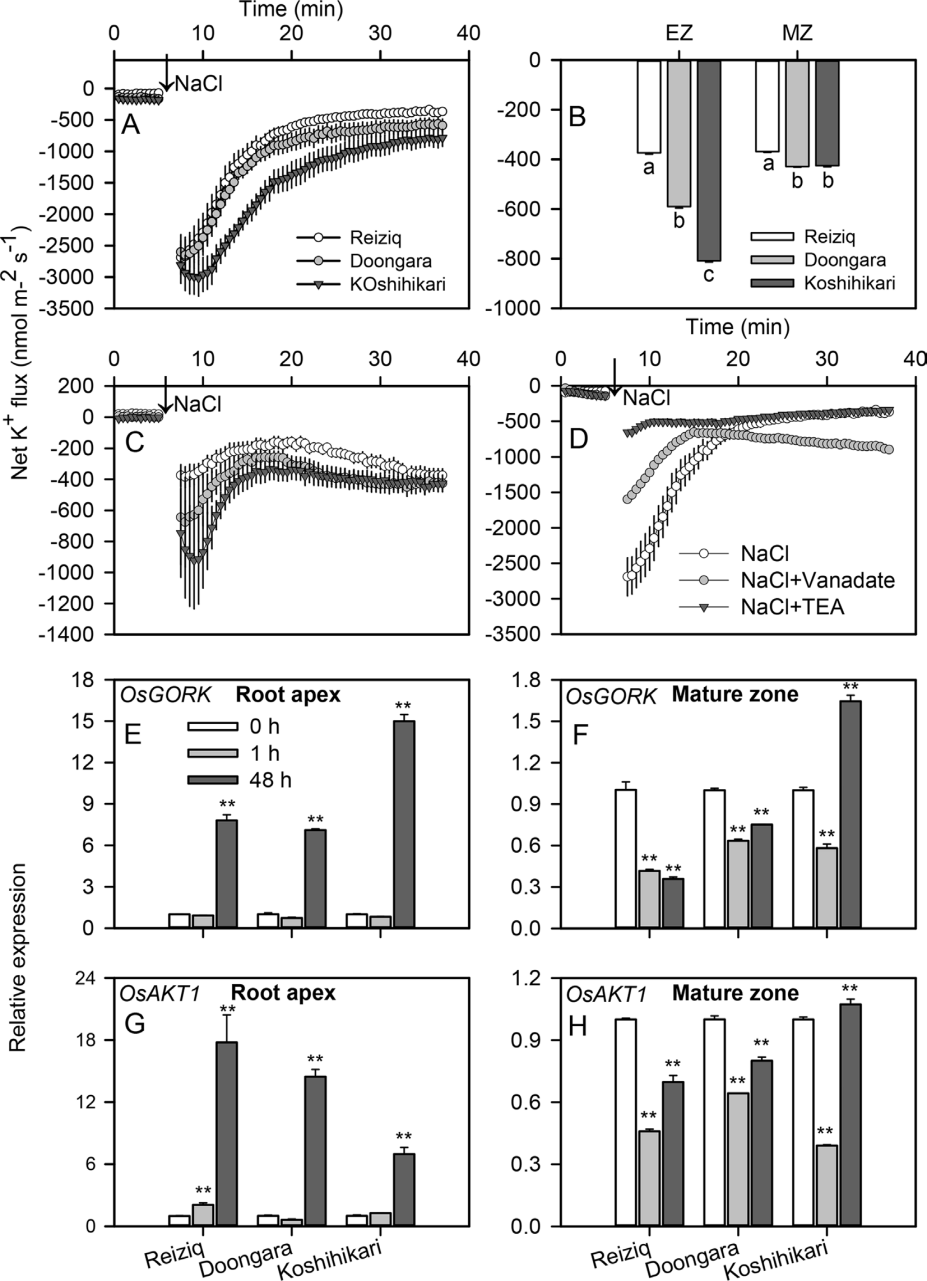
Transient net K^+^ flux kinetics measured in the three rice cultivars from the elongation **(A)** and mature **(C)** root zones in response to 100 mM NaCl stress. **(B)** Steady K^+^ flux from roots 30 min after Na^+^ addition. **(D)** Transient net K^+^ flux kinetics in response to 100 mM NaCl measured from the root elongation zone of cultivar Reiziq pretreated with 20 mM tetraethylammonium chloride for 1 h (abbreviated as TEA), a known blocker of K^+^-selective plasma membrane channels. Values are the mean ± SE (*n* = 6–8). Quantitative reverse transcription PCR (qRT-PCR) analysis of the time-dependent expression pattern of Os*GORK* **(E**, **F)**, *OsAKT1* **(G**, **H)** in the root tips **(E**, **G)**, and root mature zones **(F**, **H)** of the three cultivars. Asterisk ** indicates a significant difference between the tissues at *P* < 0.01.

Acute NaCl stress resulted in a strong net K^+^ efflux in both root elongation and mature zones (**Figures 5A, C**); this efflux was ∼3-fold higher in the elongation zone. In both root zones, peak K^+^ efflux inversely correlated with plant salinity tolerance (e.g. was highest in Koshihikari > Doongara > Reiziq). Salt-stimulated K^+^ efflux gradually recovered to the relative stable values as treatment time increased (**Figure 5C**). The steady K^+^ efflux observed in Reiziq, the most salt-tolerant cultivar, was significantly lower than that seen in the other two cultivars in both root zones. Pharmacological experiments showed that salt-induced K^+^ efflux was markedly inhibited by tetraethylammonium chloride (TEA), a general potassium channel blocker (>80% inhibition; **Figure 5D**) in the most salt-tolerant cultivar Reiziq.

*OsGORK* was significantly induced by salt stress in the three rice cultivars, in a time- and zone-dependent manner (**Figures 5E, F**). After 1 h of salt stress, *OsGORK* transcript levels did not change in elongation zone but decreased ca 2-fold in mature zone. Salinity treatment for 48 h resulted in 7 to 15-fold increase in *OsGORK* expression in elongation zone (**Figure 5E**); the expression level in the elongation zone was significantly higher than that in the mature zone (**Figures 5E, F**). This increase was highest in the salt-sensitive cultivar Koshihikari; this cultivar was also the only one showing a significant increase in *OsGORK* transcript in mature zone (**Figure 5F**). *OsAKT1* transcript levels were also induced by salt stress (6 to 18-fold upregulation) in elongation zone, with strongest induction in salt-tolerant cultivar Reiziq (**Figures 5G, H**). The expression pattern of *OsAKT1* was similar to that of *OsGORK* in root mature zones of all three cultivars.

**Figure 6:**
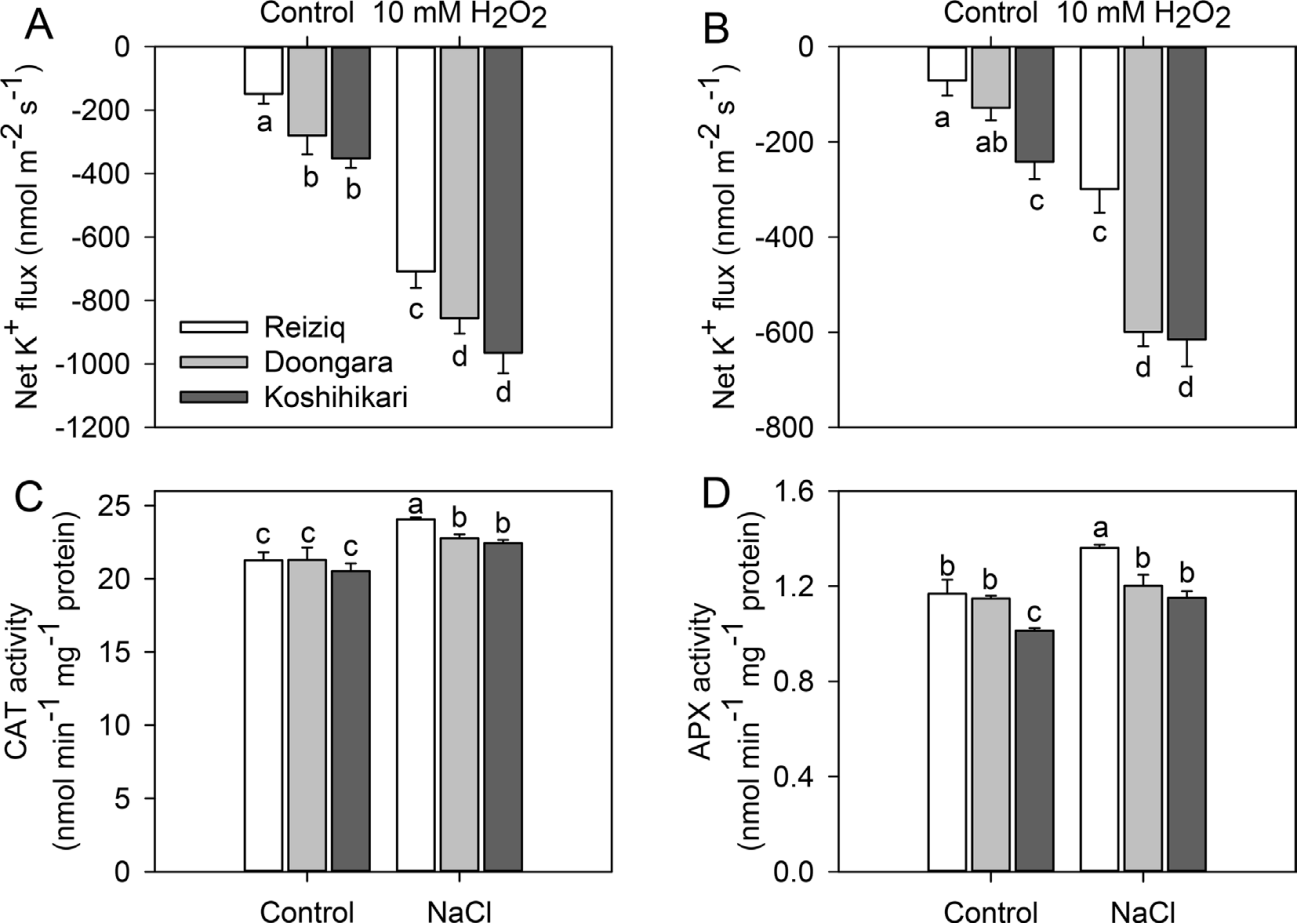
Net K^+^ fluxes from the root elongation **(A)** and mature zones **(B)** of the three rice cultivars after exposure to 10 mM H_2_O_2_ for 30 min. The changes of CAT **(C)** and APX **(D)** activity in the roots of the three rice cultivars exposed to three salinity levels for 48 h. Mean ± SE (*n* = 6). Data labeled by different letters are significantly different at *P* < 0.05.

Salinity exposure also results in a significant increase in ROS accumulation in plant tissues including roots (Munns and Tester, 2008; Bose et al., 2014b). Accordingly, we have compared a sensitivity of root epidermal cells to H_2_O_2_ treatment. H_2_O_2_ (10 mM) application increased K^+^ effluxes in both root zones, with ∼2-fold stronger responses from the elongation zones than mature zones (**Figures 6A, B**). The magnitude of H_2_O_2_-induced K^+^ efflux was Koshihikari > Doongara > Reiziq, inversely correlating with salinity tolerance. As the genotypic difference in H_2_O_2_-induced K^+^ efflux may be causally related to the amount of endogenous enzymatic antioxidants, we have quantified CAT and APX activity in roots (**Figures 6C, D**). Salinity stress had no major impact on APX activity; CAT activity increased slightly (by 10%) upon salt treatment with 100 mM NaCl (**Figure 6C**). However, difference in CAT activity was physiologically small and could hardly account for the reported ∼2.5-fold difference in the magnitude of H_2_O_2_-induced K^+^ efflux.

### Effect of Salt Stress on H^+^ Flux and Expression Levels of Genes Encoding PM H^+^-Atpase Activity in Different Root Zones

**Figure 7:**
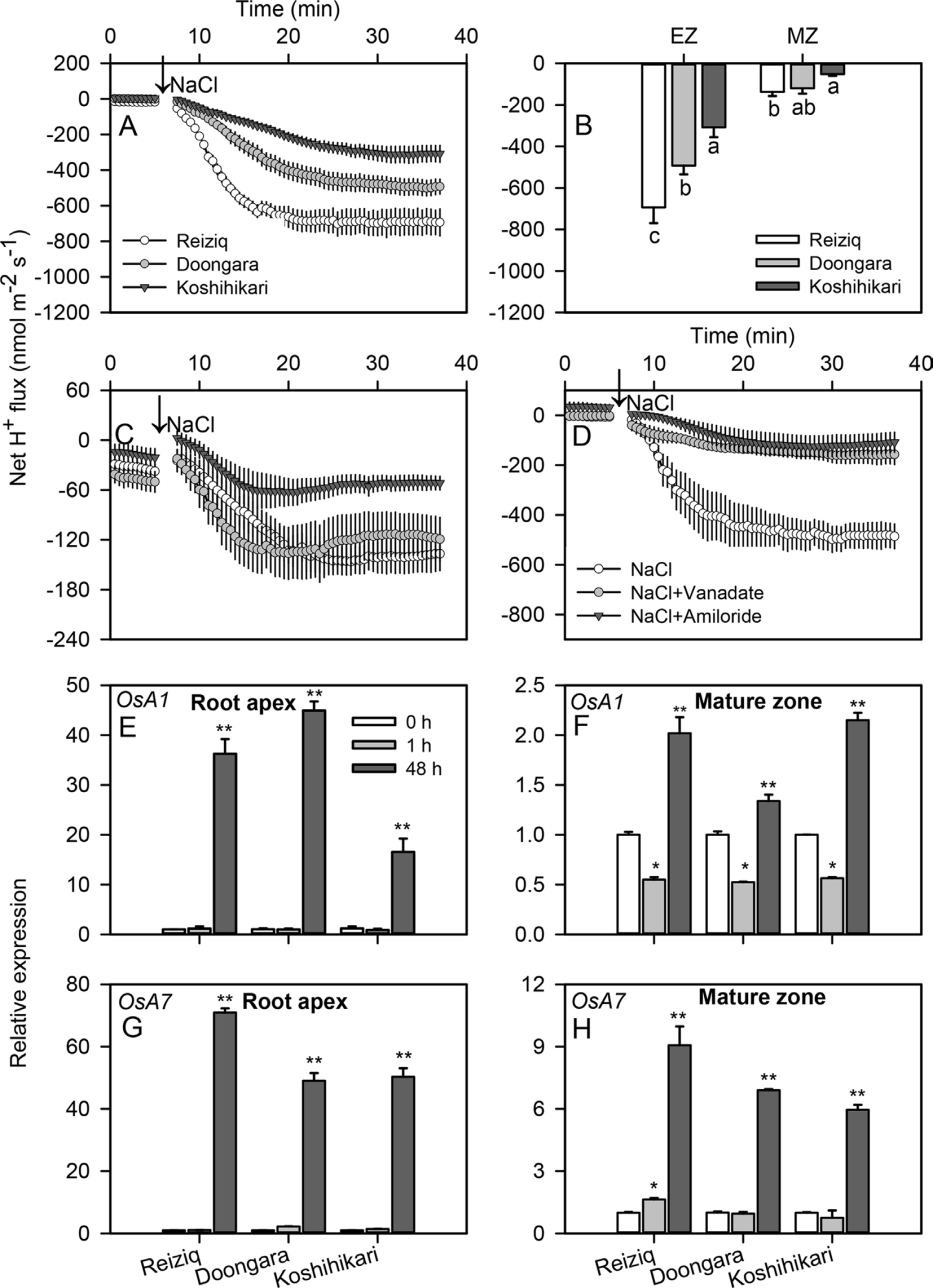
Transient net H^+^ flux kinetics measured in the three rice cultivars from the elongation **(A)** and mature **(C)** root zones in response to 100 mM NaCl stress. **(B)** Average net H^+^ flux from roots calculated over the 30 min period after 100 mM NaCl addition. **(D)** Transient net H^+^ flux kinetics in response to 100 mM NaCl treatment measured from the root elongation zone of cultivar Reiziq pretreated with inhibitors: 1 mM sodium orthovanadate (vanadate), a known blocker of PM H^+^-ATPase; 1 mM amiloride, an inhibitor of the plasma membrane Na^+^/H^+^ exchanger activity. Values are the mean ± SE (*n* = 6–8). Relative expression of the genes *OsA1* **(E**, **F)** and *OsA7* **(G**, **H)** encoding the plasma membrane H^+^-ATPase in the root tips (left panels) and mature zones (right panels) of the three cultivars. Asterisk * or ** indicate significant difference between the tissues at *P* < 0.05 or *P* < 0.01, respectively.

Higher salt load led to significant H^+^ efflux in both root zones of all cultivars (**Figures 7A, C**). Similar to K^+^ data, the magnitude of NaCl-induced H^+^ efflux was several-fold higher in the elongation zone. The magnitude of NaCl-induced H^+^ efflux correlated strongly with rice salinity tolerance and declined in the sequence Reiziq > Doongara > Koshikihari (**Figure 7B**). Pretreatment with sodium orthovanadate resulted in a major decrease in NaCl-induced H^+^ efflux (> 90% inhibition; **Figure 7D**), pointing out at the involvement of H^+^-ATPase as a major source of NaCl-induced H^+^ efflux.

To distinguish between transcriptional and functional regulation of H^+^-ATPase, we have investigated expression levels of the PM H^+^-ATPase genes *OsA1*, *OsA2*, *OsA3*, *OsA7*, and *OsA8* in root tips and mature zones under salt stress (**Figures 7E–H**; **Figure S2**). *OsA1* and *OsA7* were significantly induced after 48 h of salt stress, especially in root tips; *OsA7* expression in salt-tolerant Reiziq was about 1.5-fold higher than that in a salt-sensitive Koshihikari.

## Discussion

The present study demonstrated high genetic variability in salinity stress tolerance between rice cultivars (Reiziq > Doongara > Koshihikari) and provided some insights into mechanistic basis of this phenomenon.

### Root K^+^ Retention Is Critical for Salt Tolerance in Rice

Salt stress induces K^+^ efflux from roots (Shabala et al., 2005; Shabala et al., 2006; Sun et al., 2009; Lu et al., 2013; Lang et al., 2014; Zhao et al., 2016), and for many plant species a strong positive correlation between root K^+^ retention ability and salinity stress tolerance was reported (Wu et al., 2018a). However, the importance of this trait for rice was challenged by some authors based on the analysis of ^42^K^+^ kinetics in roots of different rice cultivars (Coskun et al., 2013). However, the above method is indirect and is based on many assumptions related to kinetics of ^42^K distribution between intracellular compartments (Lee and Clarkson, 1986; Siddiqi et al., 1991; Kronzucker et al., 1995). Here, we have utilized the MIFE K^+^-selective microelectrodes to directly measure net K^+^ efflux from rice roots in response to salinity treatment. Salt stress-induced K^+^ efflux was observed in both root elongation and mature zones (**Figures 5A, B**) and inversely correlated with salinity stress tolerance. Thus, root K^+^ retention is also a critical trait conferring salinity stress tolerance in rice as reported for other cereal species such as wheat (Cuin et al., 2008; Cuin et al., 2012) and barley (Chen et al., 2005; Chen et al., 2007a; Chen et al., 2007b; Wu et al., 2015).

### Transcriptional Regulation of GORK and AKT K^+^ Channels Under Saline Conditions May be Essential for Stress Signaling

Electrophysiological and genetic studies have demonstrated that GORK channels are considered as one of the major pathways for salinity-induced K^+^ efflux from root cells (Shabala et al., 2016; Shabala, 2017). The results of our pharmacological experiments on rice (**Figure 5D**) are consistent with these findings, with TEA, a known blocker of K^+^-permeable PM channels, blocking >80% of NaCl-induced K^+^ efflux from rice roots.

*OsGORK* transcript levels were significantly induced in root tips 48 h after salt stress in each rice cultivar tested (**Figure 5E**). Although, in the light of importance of root K^+^ retention for salinity tolerance discussed above, this seems to be counterintuitive, it is also consistent with some recent reports that salinity stress upregulated *GORK* transcript levels in roots in barley (Adem et al., 2014) and *Brassica* species (Chakraborty et al., 2016). The physiological rationale for this induction of *GORK* transcripts by salt stress may be a signaling role of K^+^ efflux as a “metabolic switch” by providing inhibition of energy-consuming biosynthesis that releases energetic molecules for defense and reparation needs (Demidchik, 2014; Shabala, 2017; Rubio et al., 2019). However, the amount of K^+^ lost for signaling purposes should not compromise the plant’s nutritional demand for this element. From our data reported here, this dilemma is resolved by at least two possible ways. First, salt stress-induced increase in *GORK* transcripts is not as pronounced in tolerant cultivars as in salt-sensitive Koshihikari (a 2-fold difference; **Figure 5E**). Second, NaCl-induced increase in *GORK* transcript level is accompanied by a concurrent increase in the amount of *OsAKT1* transcripts (**Figure 5G**). This increase is much more pronounced in tolerant varieties; as a result, the ratio in relative increase between *AKT1* and *GORK* transcript levels was 2.4 in the most tolerant cultivar Reiziq; 2.14 in less tolerant Doongara; and only 0.43 in salt-sensitive Koshihikari. Thus, a “compensation mechanism” appears to exist in rice species, that might allow plants to use root K^+^ efflux for signaling purpose without compromising plant nutritional status. The efficiency of this compensation mechanism ultimately determines the extent of salinity stress tolerance.

### Amiloride-Sensitive Na^+^/H^+^ Exchangers Play a Relatively Small Role in Na^+^ Exclusion from Rice Roots

The ability to extrude Na^+^ from roots has been widely accepted as an important determinant of salt tolerance in plant species (Munns and Tester, 2008). Rice is no exception. Compared to salt-sensitive cultivar Koshihikari, tolerant cultivars Reiziq and Doongara exhibited significantly higher Na^+^ extrusion but only in the root elongation zone (**Figure 4A**).

It is generally accepted that root Na^+^ extrusion is mediated by Na^+^/H^+^ exchanger from NHE/NHX family encoded by SOS1 (Shi et al., 2002) that is fueled by operation of the H^+^-ATPase pump. These Na^+^/H^+^ exchangers have a small conserved stretch of 14 residues in the fourth transmembrane segment, with the consensus LLPPI acting as the binding site for its inhibitor amiloride (Putney et al., 2002). Plant SOS1 exchangers also contain a region largely aligned to this conserved motif (Wu et al., 2019) and thus are sensitive to amiloride (Cuin et al., 2011; Wu et al., 2019). However, in this study, Na^+^ efflux was only partly inhibited by amiloride (28% suppression; *P* < 0.05), an inhibitor of Na^+^/H^+^ exchanger activity, and by sodium orthovanadate (37% suppression; *P* < 0.05), an inhibitor of H^+^-ATPase activity (**Figure 4B**). This is in a stark contrast to the previous findings on *Arabidopsis* (Shabala and Cuin, 2008) and wheat and barley, where Na^+^ efflux was inhibited by amiloride (> 80% inhibition) (Wu et al., 2018b; Wu et al., 2019). Also, SOS1 exchangers are predominantly located in the root apex (Shi et al., 2002), while in our study, net Na^+^ efflux was not different between elongation and mature zones in the salt-sensitive cultivar Koshihikari and was only 2-fold different in the other two (tolerant) cultivars (**Figure 4A**). Taken together, this data suggests that amiloride-sensitive Na^+^/H^+^ exchangers play a relatively small role in Na^+^ exclusion from rice roots. These findings were unexpected, as *OsSOS1* has been genetically characterized as the sole Na^+^ efflux transporter to date in rice (EI Mahi et al., 2019). Consistent with our findings, we also found that salt induced a significant increase in *OsSOS1* expression in all cultivars (∼ 20-fold in elongation zone; ∼ 6-fold in mature zone; **Figures 4C, D**). It appears that for some unknown reasons these transcriptional changes were not translated into the operation of SOS1 protein at the functional level.

In *Arabidopsis*, SOS1-mediates Na^+^/H^+^ exchange is controlled by a multifunctional protein kinase encoded by *SOS2* (Qiu et al., 2002). However, changes in *SOS2* expression did not correlate with genotypic difference in salinity stress tolerance in our cultivars (**Figure 4E**). Thus, it appears that the role of SOS signaling pathway and Na^+^ exclusion in rice differ significantly from that in *Arabidopsis* and other species. Also, it appears that findings from transcriptional studies cannot be directly extrapolated to predict operation of a specific transporter(s), calling for higher emphasis on the functional studies.

### Anatomical Changes in Rice Roots

Exposure to salinity resulted in a shrinkage and a physical damage to the pericycle in a salt-sensitive cultivar (**Figure 3**). We believe that these anatomical changes might contribute to increased Na^+^ accumulation in the shoot (and, hence, loss of salt tolerance). Amongst all root cells, the pericycle cells are considered to possess the greatest ability to sequester Na^+^ and Cl^-^ in vacuoles (Storey et al., 2003) so the loss of its integrity might come with a compromised ability for root tissue tolerance. The loss of pericycle integrity might also affect the rate of (uncontrollable) xylem Na^+^ loading. It was earlier reported that genome duplication in rice improved plants adaptability to saline conditions; to the large extent these beneficial effects were attributed to ultrastructural changes in the root anatomy and formation of the protective gap produced between the cortex cells and pericycle cells (Tu et al., 2014). The causal link between salinity-induced changes in the root anatomy, tissue-specific expression of specific ion transporters, and the modes of their operation, warrants a separate investigation.

## Conclusion

In summary, our data suggests root K^+^ retention plays a prominent role in conferring genetic variability in salinity tolerance trait in rice, and is conferred by orchestrated regulation of several mechanisms, both at transcriptional and post-translational level. At the same time, it appears that SOS signaling pathway cannot explain the above genetic variability and is less essential for root Na^+^ exclusion in rice compared with other species.

## Data Availability Statement

The datasets generated for this study will not be made publicly available n/a.

## Author Contributions

SS conceived and designed the experiments; JL performed the experiments and analyzed the data; LS, ZC, and FZ provided methodological support for experiments; QZ, GV, HM, and MX critically assessed the data; JL and SS wrote the paper.

## Funding

This work was supported by Australia-India Strategic Research Funding (AISRF48490) grant to SS. This work was supported by the Rice Industry Technology System of Henan Province (S2012-04-G02) grant to QZ. We thank Sunrice (Ricegrowers Limited) for kind supply of seeds of Australian rice varieties.

## Conflict of Interest

The authors declare that the research was conducted in the absence of any commercial or financial relationships that could be construed as a potential conflict of interest.

## Supplementary Material

Supplemental Table S1 List of primers used for qRT-PCR analysis.

Supplemental Figure S1 Tissue sampling for qRT-PCR. **(A)** Illustration of the size of the root tips and mature zones, Bar = 1 cm. **(B)** Illustration of the size of the root tips, Bar = 200 μm.

Supplemental Figure S2 Relative expression of *OsA2* **(A**, **B)**, *OsA3* **(C**, **D)** and *OsA8* **(E**, **F)** genes encoding the plasma membrane H^+^-ATPase in the root tips (left panels) and mature zones (right panels) of three rice cultivars.
