# Supplementary material for: Tissue-Specific Regulation of Na+ and K+ Transporters Explains Genotypic Differences in Salinity Stress Tolerance in Rice
Source: Front Plant Sci. 2019 Nov 1;10:1361. doi: 10.3389/fpls.2019.01361 (PMC6838216; doi:10.3389/fpls.2019.01361)
Supplement: Supplemental Table S1 — List of primers used for qRT-PCR analysis. [file Table_1.pdf]

Table S1 List of primers used for quantitative real-time PCR (qRT-PCR)

**Gene**	**Gene ID**	**Forward Primer**	**Reverse Primer**
OsSOS1	Os12g0641100	ATCAGGTGGAGGCTAGAGCA	TGACGCACTCCTTTGCAGAT
OsSOS2	Os06g0606000	AACAGAATGAGCCCGTTTGC	TCCAGTGTGTCACCAGCAAC
OsGORK	Os06g0250600	TTGTCGTCTTTTGCGGCTTG	GCTGCCGTATTCGCTACTCT
OsAKT1	Os01g0648000	ACAGCCGACAATGTGGTGAA	CGCCATGCGATCTTCTTTGG
OSA1	Os03g0689300	ACGATGCTCCAGCCCTAAA	ATGGCGGGAAATCAAACTC
OSA2	Os07g0191200	ACTGCTCAGAGGACCATCCA	TGCGATTTCGGCTCTCCTAC
OSA3	Os12g0638700	CTGTCACCAAAGGTCCTGGT	TGCAGAAATTCCCAATAGCC
OSA7	Os04g0656100	TTCCATCACCATCCGTATAGTG	AGAAGGCTTAACTCTGTCCTTC
OSA8	Os03g0100800	TTGGGCAGCCTACAAGACA	AAGGACCAACCTCTGGAACG
β-Actin	Os03g0718150	GGAAGTACAGTGTCTGGATTGGAG	TCTTGGCTTAGCATTCTTGGGT
