# Supplementary figures and images for: Tissue-Specific Regulation of Na+ and K+ Transporters Explains Genotypic Differences in Salinity Stress Tolerance in Rice
Source: Front Plant Sci. 2019 Nov 1;10:1361. doi: 10.3389/fpls.2019.01361 (PMC6838216; doi:10.3389/fpls.2019.01361)

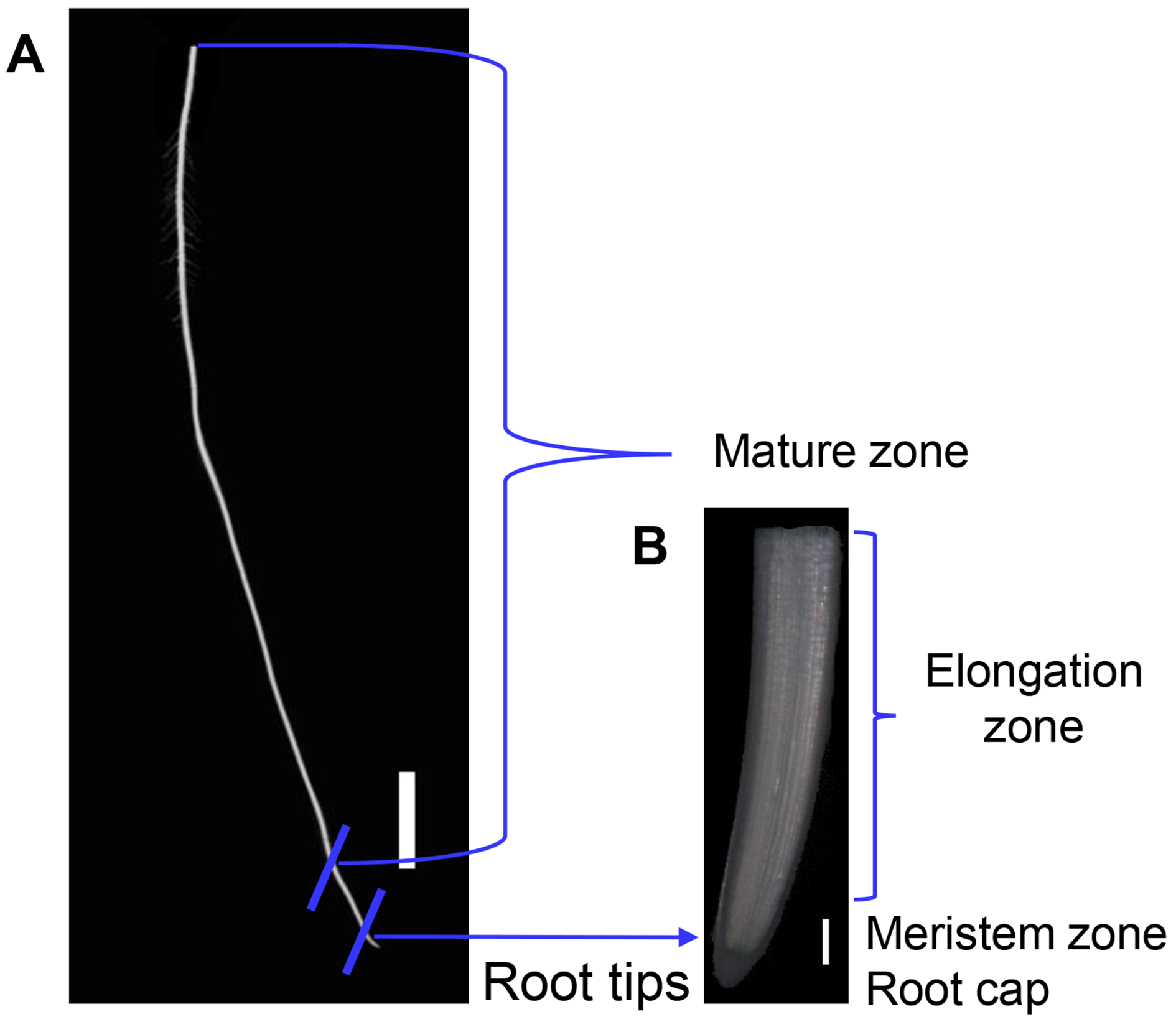

Supplement: Supplemental Figure S1 — Tissue sampling for qRT-PCR. (A) Illustration of the size of the root tips and mature zones, Bar = 1 cm. (B) Illustration of the size of the root tips, Bar = 200 μm. [file Image_1.tif]

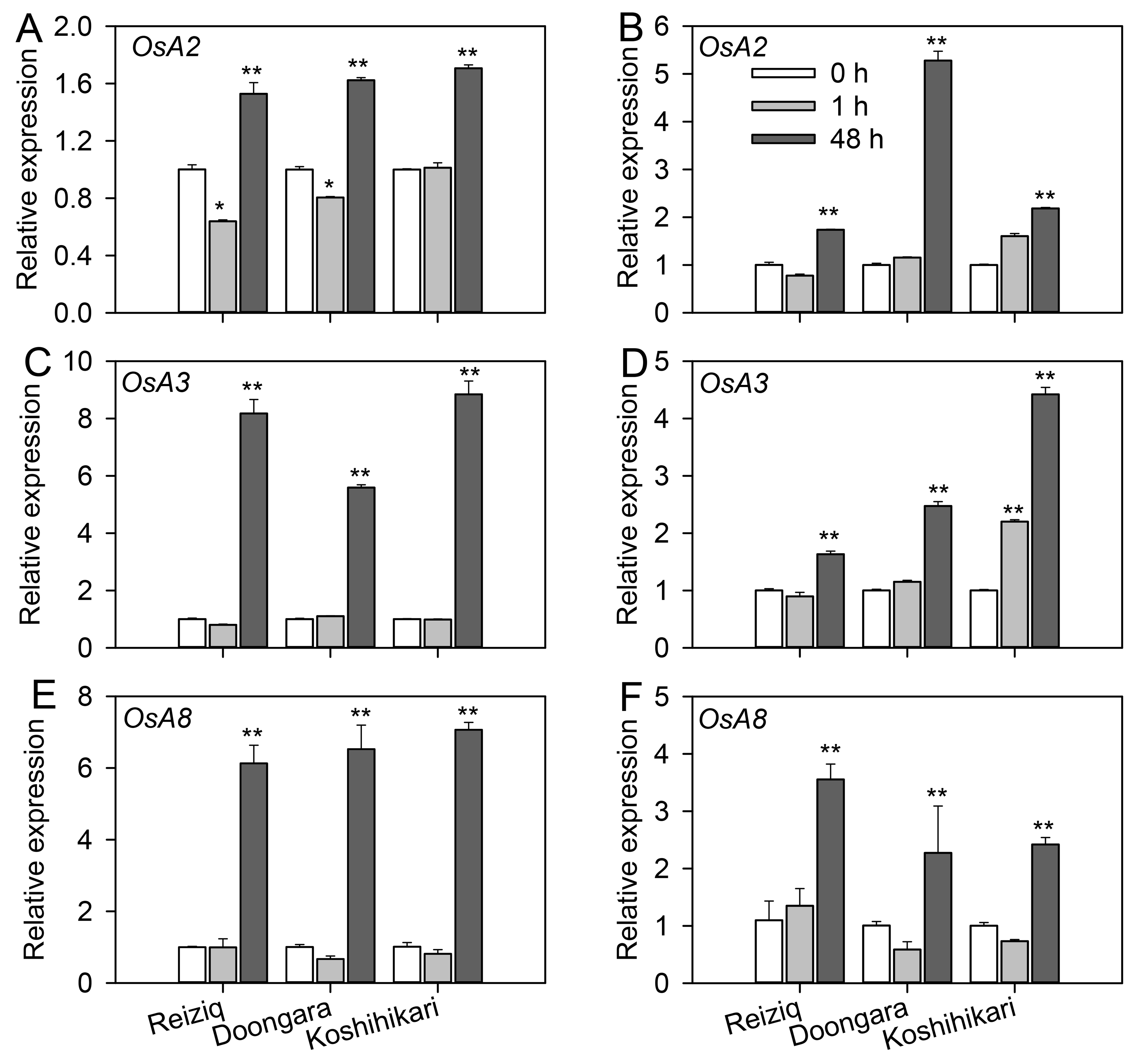

Supplement: Supplemental Figure S2 — Relative expression of OsA2 (A, B), OsA3 (C, D) and OsA8 (E, F) genes encoding the plasma membrane H+-ATPase in the root tips (left panels) and mature zones (right panels) of three rice cultivars. [file Image_2.tif]
